# The ESCRT System Is Required for Hepatitis C Virus Production

**DOI:** 10.1371/journal.pone.0014517

**Published:** 2011-01-11

**Authors:** Yasuo Ariumi, Misao Kuroki, Masatoshi Maki, Masanori Ikeda, Hiromichi Dansako, Takaji Wakita, Nobuyuki Kato

**Affiliations:** 1 Department of Tumor Virology, Okayama University Graduate School of Medicine, Dentistry, and Pharmaceutical Sciences, Okayama, Japan; 2 Department of Applied Molecular Biosciences, Graduate School of Bioagricultural Sciences, Nagoya University, Nagoya, Japan; 3 Department of Virology II, National Institute of Infectious Diseases, Tokyo, Japan; INMI, Italy

## Abstract

**Background:**

Recently, lipid droplets have been found to be involved in an important cytoplasmic organelle for hepatitis C virus (HCV) production. However, the mechanisms of HCV assembly, budding, and release remain poorly understood. Retroviruses and some other enveloped viruses require an endosomal sorting complex required for transport (ESCRT) components and their associated proteins for their budding process.

**Methodology/Principal Findings:**

To determine whether or not the ESCRT system is needed for HCV production, we examined the infectivity of HCV or the Core levels in culture supernatants as well as HCV RNA levels in HuH-7-derived RSc cells, in which HCV-JFH1 can infect and efficiently replicate, expressing short hairpin RNA or siRNA targeted to tumor susceptibility gene 101 (TSG101), apoptosis-linked gene 2 interacting protein X (Alix), Vps4B, charged multivesicular body protein 4b (CHMP4b), or Brox, all of which are components of the ESCRT system. We found that the infectivity of HCV in the supernatants was significantly suppressed in these knockdown cells. Consequently, the release of the HCV Core into the culture supernatants was significantly suppressed in these knockdown cells after HCV-JFH1 infection, while the intracellular infectivity and the RNA replication of HCV-JFH1 were not significantly affected. Furthermore, the HCV Core mostly colocalized with CHMP4b, a component of ESCRT-III. In this context, HCV Core could bind to CHMP4b. Nevertheless, we failed to find the conserved viral late domain motif, which is required for interaction with the ESCRT component, in the HCV-JFH1 Core, suggesting that HCV Core has a novel motif required for HCV production.

**Conclusions/Significance:**

These results suggest that the ESCRT system is required for infectious HCV production.

## Introduction

Hepatitis C virus (HCV) is a causative agent of chronic hepatitis, which progresses to liver cirrhosis and hepatocellular carcinoma. HCV is an enveloped virus with a positive single stranded 9.6 kb RNA genome, which encodes a large polyprotein precursor of approximately 3,000 amino acid residues. This polyprotein is cleaved by a combination of the host and viral proteases into at least 10 proteins in the following order: Core, envelope 1 (E1), E2, p7, nonstructural protein 2 (NS2), NS3, NS4A, NS4B, NS5A, and NS5B [1]. HCV Core, a highly basic RNA-binding protein, forms a viral capsid and is targeted to lipid droplets [2]–[6]. The Core is essential for infectious virion production [7]. NS5A, a membrane-associated RNA-binding phosphoprotein, is also involved in the assembly and maturation of infectious HCV particles [8], [9]. Intriguingly, NS5A is a key regulator of virion production through the phosphorylation by casein kinase II [9]. Recently, lipid droplets have been found to be involved in an important cytoplasmic organelle for HCV production [4]. Indeed, NS5A is known to colocalize with the Core on lipid droplets [5], and the interaction between NS5A and the Core is critical for the production of infectious HCV particles [3]. However, the host factor involved in HCV assembly, budding, and release remains poorly understood.

Budding is an essential step in the life cycle of enveloped viruses. Endosomal sorting complex required for transport (ESCRT) components and associated factors, such as tumor susceptibility gene 101 (TSG101, a component of ESCRT-I), charged multivesicular body protein 4b (CHMP4b, a component of ESCRT-III), and apoptosis-linked gene 2 interacting protein X (ALIX, a TSG101- and CHMP4b-binding protein), have been found to be involved in membrane remodeling events that accompany endosomal protein sorting, cytokinesis, and the budding of several enveloped viruses, such as human immunodeficiency virus type 1 (HIV-1) [10]–[12]. The ESCRT complexes I, II, and III are sequentially, or perhaps concentrically recruited to the endosomal membrane to sequester cargo proteins and drive vesicularization into the endosome. Finally, ESCRT-III recruits Vps4 (two isoforms, Vps4A and Vps4B), a member of the AAA-family of ATPase that disassembles and thereby terminates and recycles the ESCRT machinery.

Since HCV is also an enveloped RNA virus, we hypothesized that the ESCRT system might be required for HCV production. To test this hypothesis, we examined the release of HCV Core into culture supernatants from cells rendered defective for ESCRT components by RNA interference. The results provide evidence that the ESCRT system is required for HCV production.

## Materials and Methods

### Cell Culture

293FT cells (Invitrogen, Carlsbad, CA) were cultured in Dulbecco's modified Eagle's medium (DMEM; Invitrogen) supplemented with 10% fetal bovine serum (FBS). The HuH-7-derived cell line, RSc cured cells that cell culture-generated HCV-JFH1 (JFH1 strain of genotype 2a) [13] could infect and effectively replicate [14]–[16] and OR6c and OR6 cells harboring the genome-length HCV-O RNA with luciferase as a reporter were cultured in DMEM with 10% FBS as described previously [17], [18].

### Plasmid Construction

To construct pcDNA3-FLAG-Alix, a DNA fragment encoding Alix was amplified from total RNAs derived from RSc cells by RT-PCR using the follwing pairs of primers: Forward 5′-CGGGATCCAAGATGGCGACATTCATCTCGGT-3′ and reverse 5′-CCGGCGGCCGCTTACTGCTGTGGATAGTAAG-3′. The obtained DNA fragment was subcloned into *Bam*HI-*Not*I of pcDNA3-FLAG vector [19], and the nucleotide sequences were determined by Big Dye termination cycle sequencing using an ABI Prism 310 genetic analyzer (Applied Biosystems, Foster City, CA, USA). The plasmid of pJRN/3-5B was based on pJFH1 [13] and was constructed as previously described [20].

### RNA synthesis, RNA transfection, and Selection of G418-resistant cells

Plasmid pJRN/3-5B were linearized by *Xba*I and used for the RNA synthesis with the T7 MEGAScript kit (Ambion, Austin, TX). *In vitro* transcribed RNA was transfected into OR6c cells by electroporation [17], [18]. The transfected cells were selected in culture medium containing G418 (0.3 mg/ml) for 3 weeks. We referred to them as OR6c/JRN 3-5B cells.

### Immunofluorescence and Confocal Microscopic Analysis

Cells were fixed in 3.6% formaldehyde in phosphate-buffered saline (PBS) and permeabilized in 0.1% Nonidet P-40 (NP-40) in PBS at room temperature as previously described [21]. Cells were incubated with anti-HCV Core antibody (CP-9 and CP-11 mixture; Institute of Immunology, Tokyo, Japan), anti-Myc-Tag antibody (PL14; Medical & Biological Laboratories, MBL, Nagoya, Japan), anti-Alix antibody (Covalab, Villeurbanne, France), and/or anti-FLAG polyclonal antibody (Sigma, St. Louis, MO) at a 1∶300 dilution in PBS containing 3% bovine serum albumin (BSA) at 37°C for 30 min. Cells were then stained with fluorescein isothiocyanate (FITC)-conjugated anti-rabbit antibody (Jackson ImmunoResearch, West Grove, PA) or anti-Cy3-conjugated anti-mouse antibody (Jackson ImmunoResearch) at a 1∶300 dilution in PBS containing BSA at 37°C for 30 min. Lipid droplets and nuclei were stained with BODIPY 493/503 (Molecular Probes, Invitrogen) and DAPI (4′,6′-diamidino-2-phenylindole), respectively. Following extensive washing in PBS, cells were mounted on slides using a mounting media of 90% glycerin/10% PBS with 0.01% *p*-phenylenediamine added to reduce fading. Samples were viewed under a confocal laser-scanning microscope (LSM510; Zeiss, Jena, Germany).

### RNA Interference

The following siRNAs were used: human TSG101 (siGENOME SMARTpool M-003549-01-0005 and 5′-CCUCCAGUCUUCUCUCGUCUU-3′ sense, 5′-GACGAGAGAAGACUGGAGGUU-3′ antisense), human Alix/PDCD6IP (siGENOME SMARTpool M-004233-02-0005), human Vps4B (siGENOME SMARTpool M-013119-02-0005), human CHMP4b (siGENOME SMARTpool M-018075-00-0005), and siGENOME Non-Targeting siRNA Pool#1 (D-001206-13-05) (Dharmacon, Thermo Fisher Scientific, Waltham, MA) as a control. siRNAs (50 nM final concentration) were transiently transfected into either RSc cells [14]–[16] or OR6 cells [17], [18] using Oligofectamine (Invitrogen) according to the manufacturer's instructions. Oligonucleotides with the following sense and antisense sequences were used for the cloning of short hairpin (sh) RNA-encoding sequences against TSG101, Alix, Vps4B, or CHMP4b in lentiviral vector: TSG101i, 5′-GATCCCC GGAGGAAATGGATCGTGCCTTCAAGAGAGGCACGATCCATTTCCTCCTTTTTGGAAA-3′ (sense), 5′-AGCTTTTCCAAAAAGGAGGAAATGGATCGTGCCTCTCTTGAAGGCACGATCCATTTCCTCCGGG-3′ (antisense); Alixi, 5′-GATCCCC GGAGGTGTTCCCTGTCTTGTTCAAGAGACAAGACAGGGAACACCTCCTTTTTGGAAA-3′ (sense), 5′-AGCTTTTCCAAAAAGGAGGTGTTCCCTGTCTTGTCTCTTGAACAAGACAGGGAACACCTCCGGG-3′ (antisense); Vps4Bi, 5′-GATCCCC GGAGAATCTGATGATCCTGTTCAAGAGACAGGATCATCAGATTCTCCTTTTTGGAAA-3′ (sense), 5′-AGCTTTTCCAAAAAGGAGAATCTGATGATCCTGTCTCTTGAACAGGATCATCAGATTCTCCGGG-3′ (antisense); CHMP4bi, 5′-GATCCCC GAGGAGGACGACGACATGATTCAAGAGATCATGTCGTCGTCCTCCTCTTTTTGGAAA-3′ (sense), 5′-AGCTTTTCCAAAAAGAGGAGGACGACGACATGATCTCTTGAATCATGTCGTCGTCCTCCTCGGG-3′ (antisense); Broxi, 5′-GATCCCCGGATGACAGTACTAAACCCTTCAAGAGAGGGTTTAGTACTGTCATCCTTTTTGGAAA-3′ (sense), 5′-AGCTTTTCCAAAAAGGATGACAGTACTAAACCCTCTCTTGAAGGGTTTAGTACTGTCATCCGGG-3′ (antisense). The oligonucleotides above were annealed and subcloned into the *Bgl*II-*Hin*dIII site, downstream from an RNA polymerase III promoter of pSUPER [22], to generate pSUPER-TSG101i, pSUPER-Alixi, pSUPER-Vps4Bi, and pSUPER-CHMP4bi, respectively. To construct pLV-TSG101i, pLV-Alixi, pLV-Vps4Bi, and pLV-CHMP4bi, the *Bam*HI-*Sal*I fragments of the corresponding pSUPER plasmids were subcloned into the *Bam*HI-*Sal*I site of pRDI292 [23], an HIV-1-derived self-inactivating lentiviral vector containing a puromycin resistant marker allowing for the selection of transduced cells, respectively.

### Lentiviral Vector Production

The vesicular stomatitis virus (VSV)-G-pseudotyped HIV-1-based vector system has been described previously [24]–[26]. The lentiviral vector particles were produced by transient transfection of the second-generation packaging construct pCMV-ΔR8.91 [24]–[26] and the VSV-G-envelope-expressing plasmid pMDG2 as well as pRDI292 into 293FT cells with FuGene6 (Roche Diagnostics, Basel, Switzerland).

### HCV Infection Experiments

The supernatants was collected from cell culture-generated HCV-JFH1 [13]-infected RSc cells [14]–[16] at 5 days post-infection and stored at −80°C after filtering through a 0.45 µm filter (Kurabo, Osaka, Japan) until use. For infection experiments with HCV-JFH1 virus, RSc cells (1×10^5^ cells/well) were plated onto 6-well plates and cultured for 24 hours (hrs). Then, we infected the cells with 50 µl (equivalent to a multiplicity of infection [MOI] of 0.1) of inoculum. The culture supernatants were collected and the levels of HCV Core were determined by enzyme-linked immunosorbent assay (ELISA) (Mitsubishi Kagaku Bio-Clinical Laboratories, Tokyo, Japan). Total RNA was isolated from the infected cellular lysates using RNeasy mini kit (Qiagen, Hilden, Germany) for quantitative RT-PCR analysis of intracellular HCV RNA. The infectivity of HCV in the culture supernatants was determined by a focus-forming assay at 48 hrs post-infection. The HCV infected cells were detected using anti-HCV Core antibody (CP-9 and CP-11). Intracellular HCV infectivity was determined by a focus-forming assay at 48 hrs post-inoculation of lysates by repeated freeze and thaw cycles (three times).

### Quantitative RT-PCR Analysis

The quantitative RT-PCR analysis for HCV RNA was performed by real-time LightCycler PCR (Roche) as described previously [17], [18]. We used the following forward and reverse primer sets for the real-time LightCycler PCR: TSG101, 5′-ATGGCGGTGTCGGAGAGCCA-3′ (forward), 5′-AACAGGTTTGAGATCTTTGT-3′ (reverse); Alix, 5′-ATGGCGACATTCATCTCGGT-3′ (forward), 5′-TACTGGGCCTGCTCTTCCCC-3′ (reverse); Vps4B, 5′-ATGTCATCCACTTCGCCCAA-3′ (forward), 5′-ATACTGCACAGCATGCTGAT-3′ (reverse); CHMP4b, 5′-ATGTCGGTGTTCGGGAAGCT-3′ (forward), 5′-ATCTCTTCCGTGTCCCGCAG-3′ (reverse); Brox, 5′-ATGACCCATTGGTTTCATAG-3′ (forward), 5′-CCTGGATGACCTCAAGTCAT-3′ (reverse); β-actin, 5′-TGACGGGGTCACCCACACTG-3′ (forward), 5′-AAGCTGTAGCCGCGCTCGGT-3′ (reverse); and HCV-JFH1, 5′-AGAGCCATAGTGGTCTGCGG-3′ (forward), 5′-CTTTCGCAACCCAACGCTAC-3′ (reverse).

### MTT Assay

Cells (5×10^3^ cells/well) were plated onto 96-well plates and cultured for 24, 48 or 72 hrs, then, subjected to the colorimetric 3-(4,5-dimethylthiazol-2-yl)-2,5-diphenyltetrazolium bromid (MTT) assay according to the manufacturer's instructions (Cell proliferation kit I, Roche). The absorbance was read using a microplate reader (Multiskan FC, Thermo Fisher Scientific) at 550 nm with a reference wavelength of 690 nm.

### Renilla Luciferase (RL) Assay

OR6 cells (1.5×10^4^ cells/well) [17], [18] were plated onto 24-well plates and cultured for 24 hrs. The cells were transfected with siRNAs (50 nM) using Oligofectamine and incubated for 72 hrs, then, subjected to the RL assay according to the manufacturer's instructions (Promega, Madison, WI). A lumat LB9507 luminometer (Berthold, Bad Wildbad, Germany) was used to detect RL activity.

### Western Blot Analysis

Cells (2×10^5^ cells/well) were plated onto 6-well plates and cultured for 24 or 48 hrs. Cells were lysed in buffer containing 50 mM Tris-HCl (pH 8.0), 150 mM NaCl, 4 mM EDTA, 1% NP-40, 0.1% sodium dodecyl sulfate (SDS), 1 mM dithiothreitol (DTT) and 1 mM phenylmethylsulfonyl fluoride (PMSF). Supernatants from these lysates were subjected to SDS-polyacrylamide gel electrophoresis, followed by immunoblot analysis using anti-TSG101 antibody (BD Transduction Laboratories, San Jose, CA), anti-Alix antibody, anti-Vps4B antibody (Abnova, Taipei, Taiwan) (A302-078A; Bethyl Laboratories, Montgomery, TX), anti-CHMP4B antibody (sc-82557; Santa Cruz Biotechnology, Santa Cruz, CA), anti-HCV Core antibody, anti-β-actin antibody (Sigma), anti-Myc-Tag antibody, anti-FLAG antibody (M2; Sigma), anti-Chk2 antibody (DCS-273; MBL), anti-heat shock protein (HSP) 70 antibody (BD), Living Colors A.v. monoclonal antibody (JL-8; Clontech, Mountain View, CA), anti-HCV NS5A monoclonal antibody (no. 8926; a generous gift from A Takamizawa, The Research Foundation for Microbial Diseases of Osaka University, Japan), or anti-HCV NS5A polyclonal antibody (a generous gift from K Shimotohno, Chiba Institute of Technology, Chiba, Japan).

### Immunoprecipitation Analysis

Cells were lysed in buffer containing 10 mM Tris-HCl (pH 8.0), 150 mM NaCl, 1% NP-40, 1 mM PMSF, and protease inhibitor cocktail containing 104 µM 4-(2-aminoethyl)benzenesulfonyl fluoride hydrochloride, 80 nM aprotinin, 2.1 µM leupeptin, 3.6 µM bestatin, 1.5 µM pepstatin A, and 1.4 µM E-64 (Sigma). Lysates were pre-cleaned with 30 µl of protein-G-Sepharose (GE Healthcare Bio-Sciences). Pre-cleaned supernatants were incubated with 5 µl of Living Colors A.v. monoclonal antibody or anti-FLAG antibody at 4°C for 1 hr. Following absorption of the precipitates on 30 µl of protein-G-Sepharose resin for 1 hr, the resin was washed four times with 700 µl lysis buffer. Proteins were eluted by boiling the resin for 5 min in 1× Laemmli sample buffer. The proteins were then subjected to SDS-PAGE, followed by immunoblotting analysis using either anti-FLAG antibody, Living Colors A.v. monoclonal antibody or anti-HCV Core antibody.

### Statistical Analysis

Statistical comparison of the infectivity of HCV in the culture supernatants between the knockdown cells and the control cells was performed using the Student's *t*-test. *P* values of less than 0.05 were considered statistically significant. All error bars indicate standard deviation.

## Results

### The ESCRT system is required for HCV production

**Figure 1:**
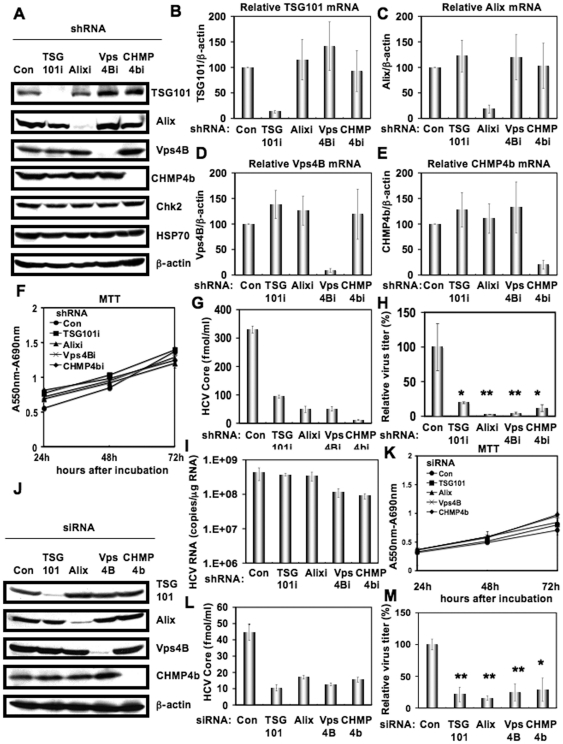
ESCRT components are required for the infectious HCV production. (A) Inhibition of TSG101, Alix, Vps4B, or CHMP4b protein expression by shRNA-producing lentiviral vectors. The results of the Western blot analysis of cellular lysates with anti-TSG101, anti-Alix, anti-Vps4B, anti-CHMP4b, anti-Chk2, anti-HSP70, or anti-β-actin antibody in RSc cells expressing shRNA targeted to TSG101 (TSG101i), Alix (Alixi), Vps4B (Vps4Bi), or CHMP4b (CHMP4bi) as well as in RSc cells transduced with a control lentiviral vector (Con) are shown. Real-time LightCycler RT-PCR for TSG101 (B), Alix (C), Vps4B (D), or CHMP4b mRNA (E) was performed as well as for β-actin mRNA in triplicate. Each mRNA level was calculated relative to the level in RSc cells transduced with a control lentiviral vector (Con) which was assigned as 100%. Error bars in this panel and other figures indicate standard deviations. (F) MTT assay of each knockdown RSc cells at the indicated time. (G) The levels of HCV Core in the culture supernatants from the stable knockdown RSc cells 97 h after inoculation of HCV-JFH1 at an MOI of 0.1 were determined by ELISA. Experiments were done in triplicate and columns represent the mean Core protein levels. (H) The infectivity of HCV in the culture supernatants from the stable knockdown RSc cells 97 hrs after inoculation of HCV-JFH1 at an MOI of 0.1 was determined by a focus-forming assay at 48 hrs post-infection. Experiments were done in triplicate and each virus titer was calculated relative to the level in RSc cells transduced with a control lentiviral vector (Con) which was assigned as 100%. Asterisks indicate significant differences compared to the control treatment. *P<0.05; **P<0.01. (I) The level of intracellular genome-length HCV-JFH1 RNA in the cells at 97 hrs post-infection was monitored by real-time LightCycler RT-PCR. Results from three independent experiments are shown. (J) Inhibition of TSG101, Alix, Vps4B, or CHMP4b protein expression by 72 hrs after transient transfection of RSc cells with a pool of control siRNA (Con) or a pool of siRNA specific for Alix, Vps4B, or CHMP4b (50 nM). The results of the Western blot analysis of cellular lysates with anti-TSG101, anti-Alix, anti-Vps4B, anti-CHMP4b, or anti-β-actin antibody is shown. (K) MTT assay of each knockdown RSc cells at the indicated time. (L) The levels of HCV Core in the culture supernatants were determined by ELISA 24 hrs after inoculation of HCV-JFH1. RSc cells were transiently transfected with a pool of control siRNA (Con) or a pool of siRNA specific for TSG101, Alix, Vps4B, or CHMP4b (50 nM). At 48 hrs after transfection, the cells were inoculated with HCV-JFH1 at an MOI of 5 and incubated for 2 hrs. Then, culture medium was changed and incubated for 22 hrs. Experiments were done in triplicate and each Core level was calculated relative to the level in the culture supernatants from the control cells and indicated below. (M) The infectivity of HCV in the culture supernatants from the transient knockdown RSc cells 24 hrs after inoculation of HCV-JFH1 at an MOI of 5 was determined by a focus-forming assay at 48 hrs post-infection. Experiments were done in triplicate and each virus titer was calculated relative to the level in RSc cells transfected with a control siRNA (Con) which was assigned as 100%. Asterisks indicate significant differences compared to the control treatment. *P<0.05; **P<0.01.

**Figure 2:**
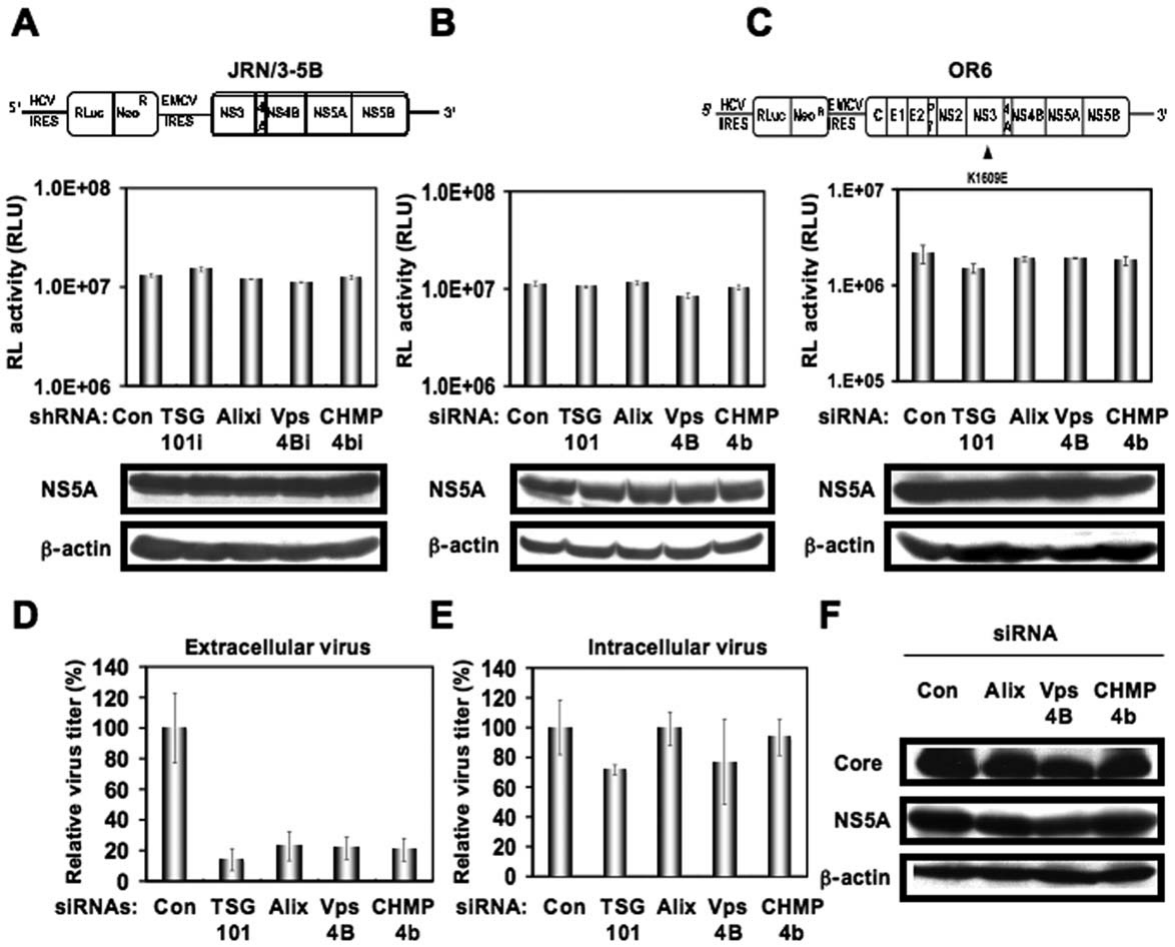
ESCRT system is not required for HCV RNA replication and assembly of intracellular infectious HCV. (A) Schematic gene organization of subgenomic JFH1 (JRN/3-5B) RNA encoding *Renilla* luciferase gene. Renilla luciferase gene (RLuc) is depicted as a box and is expressed as a fusion protein with Neo. The HCV RNA replication level in each ESCRT knockdown OR6c JRN/3-5B cells by lentiviral vector-mediated RNA interference (shRNA) was monitored by RL assay. The RL activity (RLU) is shown. The results shown are means from three independent experiments. (B) 72 hrs after the transfection of OR6c JRN/3-5B polyclonal cells with each of the siRNA (50 nM), the HCV RNA replication level was monitored by RL assay as described in (A). (C) Schematic gene organization of genome-length HCV-O RNA encoding *Renilla* luciferase gene. The position of an adaptive mutation, K1609E, is indicated by a triangle. 72 hrs after the transfection of OR6 cells with each of the siRNA (50 nM), the HCV RNA replication level was monitored by RL assay as described in (A). (D) The infectivity of HCV in the culture supernatants from the transient knockdown RSc cells 24 hrs after inoculation of HCV-JFH1 at an MOI of 2 was determined by a focus-forming assay at 48 hrs post-infection. Experiments were done in triplicate and each virus titer was calculated relative to the level in RSc cells transfected with a control siRNA (Con) which was assigned as 100%. (E) Intracellular HCV infectivity was determined by a focus-forming assay at 48 hrs post-inoculation of lysates by repeated freeze and thaw cycles as described in (D). (F) RSc cells were transiently transfected with a pool of control siRNA (Con) or a pool of siRNA specific for Alix, Vps4B, or CHMP4b (50 nM). At 24 hrs after the transfection, the cells were inoculated with HCV-JFH1 at an MOI of 0.2 and incubated for 48 hrs. Then, culture medium was changed and incubated for 24 hours. Western blotting of cell lysates 72 hrs post-infection with anti-β-actin, anti-HCV NS5A, or anti-HCV Core antibody is shown.

To investigate the potential role(s) of the ESCRT system in the HCV life cycle, we first used lentiviral vector-mediated RNA interference to stably knockdown the ESCRT components, including TSG101, Alix, Vps4B, or CHMP4b in HuH-7-derived RSc cured cells that cell-culture-generated HCVcc (HCV-JFH1, genotype 2a) [13] could infect and effectively replicate [14]–[16]. We used puromycin-resistant pooled cells 10 days after the lentiviral transduction in all experiments. Western blot and real-time LightCycler RT-PCR analyses for TSG101, Alix, Vps4B, or CHMP4b demonstrated a very effective knockdown of each ESCRT component in RSc cells transduced with lentiviral vectors expressing the corresponding shRNAs (Fig. 1A–E). Importantly, we noticed that the depletion of ESCRT components did not affect the levels of several cellular proteins, including HSP70, Chk2, and b-actin (Fig. 1A). To test the cell toxicity of each shRNA, we examined colorimetric MTT assay. In this context, we demonstrated that the shRNAs did not affect the cell viabilities (Fig. 1F). We next examined the levels of HCV Core and the infectivity of HCV in the culture supernatants as well as the level of HCV RNA in the TSG101, Alix, Vps4B, or CHMP4b stable knockdown RSc cells 97 h after HCV-JFH1 infection at an MOI of 0.1. The results showed that the release of HCV Core into the culture supernatants was significantly suppressed in these knockdown cells after HCV-JFH1 infection (Fig. 1G). Importantly, the infectivity of HCV in the culture supernatants was also significantly suppressed in these knockdown cells (Fig. 1H), while the RNA replication of HCV-JFH1 was not affected in the TSG101 or Alix knockdown cells and was somewhat decreased in the Vps4B and CHMP4b knockdown cells (Fig. 1I). This suggested that the ESCRT system is associated with infectious HCV production. To further confirm whether or not the ESCRT system is involved in HCV production, we analyzed the single-round HCV replication. For this, we used RSc cells transiently transfected with a pool of siRNAs specific for TSG101, Alix, Vps4B, or CHMP4b as well as a pool of control siRNAs (Con) following HCV infection. In spite of very effective knockdown of each ESCRT component (Fig. 1J), we demonstrated that the siRNAs did not affect the cell viabilities by MTT assay (Fig. 1K). Consistent with our finding using the stable knockdown cells, we observed that the release of HCV Core or the infectivity of HCV into the culture supernatants was significantly suppressed in these transient knockdown cells 24 hrs after HCV-JFH1 infection (Fig. 1L and 1M). Furthermore, we examined the effect of siRNA specific for TSG101, Alix, Vps4B, or CHMP4b in HCV RNA replication using the subgenomic JFH1 replicon, JRN/3-5B, encoding *Renilla* luciferase gene for monitoring the HCV RNA replication in HuH-7-derived OR6c JRN/3-5B cells (Fig. 2A and 2B) or an OR6 assay system, which was developed as a luciferase reporter assay system for monitoring genome-length HCV RNA replication (HCV-O, genotype 1b) in HuH-7-derived OR6 cells (Fig. 2C) [17], [18]. The results showed that these siRNAs could not affect HCV RNA replication as well as the levels of intracellular NS5A proteins (Fig. 2A–C). Although we have demonstrated that the ESCRT system is required for production of extracellular infectious HCV particles, it is not clear whether or not these findings are associated with the assembly of intracellular infectious particles. To test this point, infectivity of intracellular infectious particles was analyzed following lysis of HCV-JFH1-infected knockdown cells by repetitive freeze and thaw. Consequently, we did not observe any significant effects of siRNAs on the accumulation of intracellular infectious HCV-JFH1, while the accumulation of extracellular HCV was significantly suppressed in these knockdown cells (Fig. 2D and 2E), indicating that inhibition of the ESCRT system does not block the accumulation of intracellular infectious HCV particles. Furthermore, Western blot analysis of cell lysates demonstrated that the level of intracellular HCV Core and NS5A was not affected in these knockdown cells 72 hrs post-infection (Fig. 2F). Thus, we conclude that the ESCRT system is not required for the assembly of infectious particles but the ESCRT system is required for late step of HCV production.

### HCV Core can target into lipid droplets in the ESCRT knockdown cells

**Figure 3:**
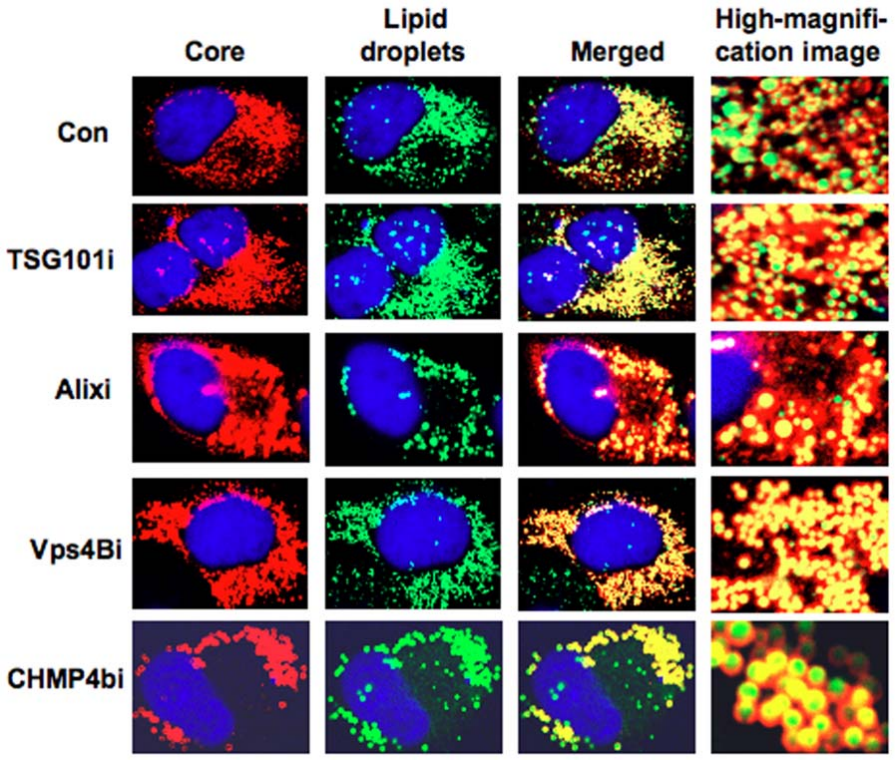
HCV Core is targeted to lipid droplets even in the ESCRT knockdown cells. The RSc cells transduced with a control lentiviral vector (Con), the TSG101 knockdown (TSG101i), the Alix knockdown (Alixi), the Vps4B knockdown (Vps4Bi), or the CHMP4b knockdown (CHMP4bi) cells were infected with HCV-JFH1. Cells were fixed 60 hrs post-infection and were then examined by confocal laser scanning microscopy. Cells were stained with anti-HCV Core (CP-9 and CP-11 mixture) and were then visualized with Cy3 (red). Lipid droplets and nuclei were stained with BODIPY 493/503 (green) and DAPI (blue), respectively. Images were visualized using confocal laser scanning microscopy. Colocalization is shown in yellow (Merged).

Since lipid droplets have been shown to be involved in an important cytoplasmic organelle for HCV production [4], we performed immunofluorescence and confocal microscopic analyses to determine whether or not HCV Core misses localization into lipid droplets in the ESCRT knockdown cells. We found that the Core was targeted into lipid droplets even in TSG101 knockdown, Alix knockdown, Vps4B knockdown, or CHMP4b knockdown RSc cells as well as in the control RSc cells after HCV infection (Fig. 3). This suggests that the ESCRT system plays a role in the late step after the Core is targeted into lipid droplets in the HCV life cycle.

### HCV Core interacts with CHMP4b

**Figure 4:**
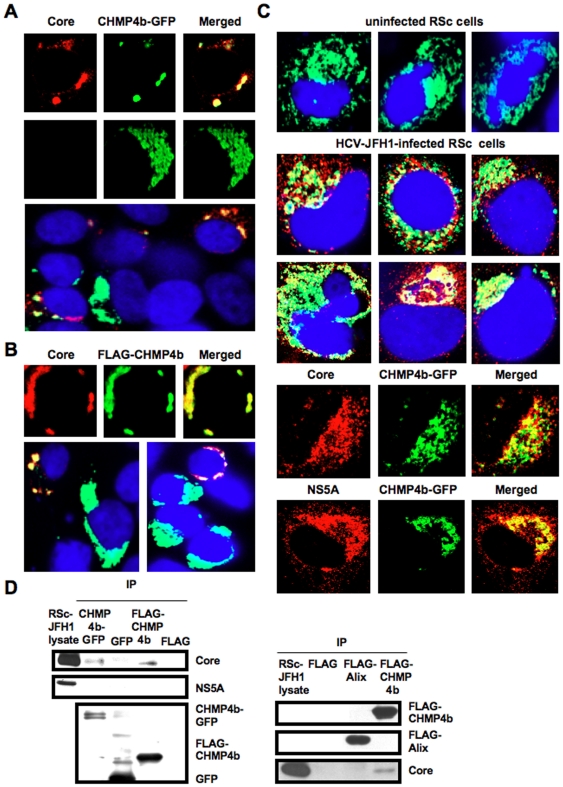
HCV Core interacts with CHMP4b. (A–C) HCV Core colocalizes with CHMP4b. 293FT cells cotransfected with 100 ng of pcDNA3/core (JFH1) and either 100 ng of pCHMP4b-GFP [39] (A) or pFLAG-CHMP4b [39] (B) were examined by confocal laser scanning microscopy. Cells were stained with anti-HCV Core and anti-FLAG polyclonal antibody and were then visualized with FITC (FLAG-CHMP4b) or Cy3 (Core). Images were visualized using confocal laser scanning microscopy. The right panels exhibit the two-color overlay images (Merged). Colocalization is shown in yellow. (C) The Core or NS5A partially colocalizes with CHMP4b in HCV-JFH1-infected RSc cells. RSc cells transfected with 100 ng of pCHMP4b-GFP were infected with HCV-JFH1. Cells were fixed 60 hrs post-infection and were then examined by confocal laser scanning microscopy as shown in panel (A). (D) HCV Core binds to CHMP4b. 293FT cells transfected with 4 µg of pCHMP4b-GFP, pEGFP C3 (Clontech), pcDNA3-FLAG, pcDNA3-FLAG-Alix or pFLAG-CHMP4b and RSc cells 5 days after inoculation of HCV-JFH1 at an MOI of 4 were lysed. The mixtures of these lysates were immunoprecipitated with either anti-FLAG or Living Colors A.v. monoclonal antibody (anti-GFP antibody), followed by immunoblot analysis using anti-HCV Core, anti-HCV NS5A, anti-FLAG, and/or Living Colors A.v. monoclonal antibody.

**Figure 5:**
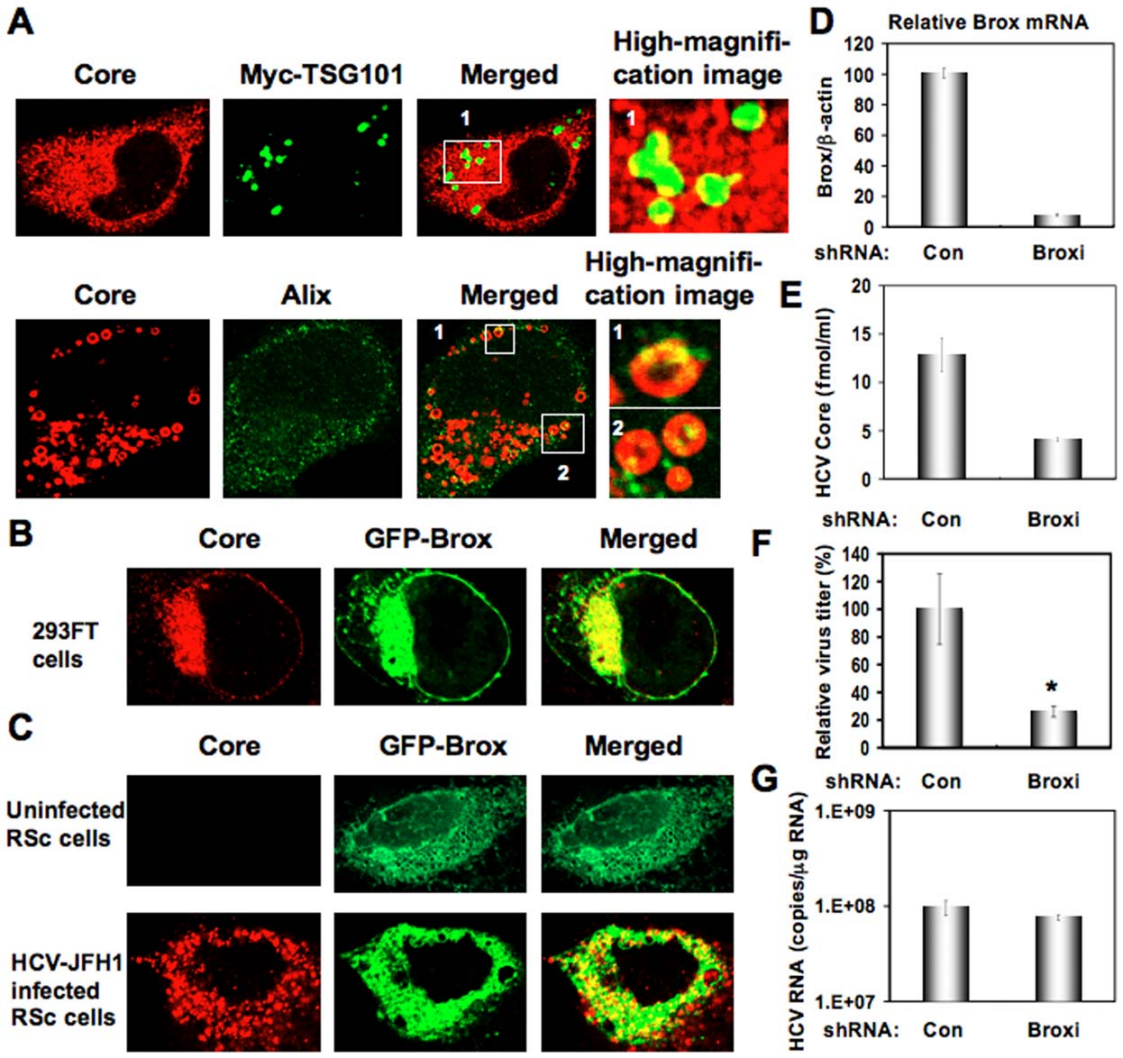
Brox is required for HCV life cycle. (A) Subcellular localization of Myc-tagged TSG101 in HCV-JFH1-infected RSc cells. RSc cells transfected with 100 ng of pBj-Myc-TSG101 [40] were infected with HCV-JFH1. Cells were fixed 60 hrs post-infection and were then examined by confocal laser scanning microscopy as shown in Fig. 3. High magnification image of area 1 is shown. Subcellular localization of endogenous Alix in HCV-JFH1-infected RSc cells 60 hrs post-infection. Cells were stained with anti-Alix and anti-HCV Core antibodies and were examined by confocal laser scanning microscopy. High magnification images of area 1 and area 2 are shown. (B) HCV Core partially colocalizes with Brox. 293FT cells cotransfected with 100 ng of pcDNA3/core (JFH1) and 100 ng of pmGFP-Brox^WT^ [27] were examined by confocal laser scanning microscopy. (C) The HCV Core partially colocalizes with Brox in HCV-JFH1-infected RSc cells. RSc cells transfected with 100 ng of pmGFP-Brox^WT^ were infected with HCV-JFH1. Cells were fixed 60 hrs post-infection and were then examined by confocal laser scanning microscopy. (D) Inhibition of Brox mRNA expression by the shRNA-producing lentiviral vector. Real-time LightCycler RT-PCR for Brox was performed as well as for β-actin mRNA in triplicate. Each mRNA level was calculated relative to the level in RSc cells transduced with a control lentiviral vector (Con) which was assigned as 100%. (E) The levels of HCV Core in the culture supernatants from the Brox knockdown RSc cells (Broxi) 72 hrs after inoculation of HCV-JFH1 were determined by ELISA. (F) The infectivity of HCV in the culture supernatants was determined by a focus-forming assay at 48 hrs post-infection. Experiments were done in triplicate and each virus titer was calculated relative to the level in RSc cells transduced with a control lentiviral vector (Con) which was assigned as 100%. Asterisks indicate significant differences compared to the control treatment. *P<0.05; **P<0.01. (G) The levels of intracellular genome-length HCV-JFH1 RNA in the cells used in (E) were monitored by real-time LightCycler RT-PCR.

To determine whether or not HCV Core can interact with ESCRT component(s), we examined their subcellular localization by confocal laser scanning microscopy. Consequently, the Core mostly colocalized with CHMP4b-green fluorescent protein (GFP) or FLAG-tagged CHMP4b in the perinuclear region of 293FT cells coexpressing them (Fig. 4A and 4B), while the CHMP4b-GFP alone was slightly diffused in the cytoplasm (Fig. 4A), indicating the recruitment of CHMP4b in the Core-expressing area. Importantly, we observed similar partial colocalization in HCV-JFH1-infected RSc cells expressing CHMP4b-GFP (Fig. 4C), whereas the CHMP4b-GFP alone was diffused in the cytoplasm in the uninfected RSc cells (Fig. 4C), suggesting the interaction of HCV Core with CHMP4b. Unfortunately, we failed to observe endogenous CHMP4b using several commercially available anti-CHMP4b antibody (data not shown). Consistent with a previous report that interaction between HCV Core and NS5A is critical for HCV production [3], we found the partial colocalization of NS5A with CHMP4b-GFP as well as the colocalization of Core with CHMP4b-GFP in HCV-JFH1-infected RSc cells (Fig. 4C). Then, we examined whether or not HCV Core can bind to CHMP4b by immunoprecipitation analysis. 293FT cells transfected with 4 mg of pCHMP4b-GFP, pEGFP C3 (Clontech), pcDNA3-FLAG [19], pcDNA3-FLAG-Alix or pFLAG-CHMP4b and RSc cells 5 days after inoculation of HCV-JFH1 at an MOI of 4 were lysed and performed immunoprecipitation of lysate mixtures of HCV-JFH1-infected RSc cells and 293FT cells expressing CHMP4b-GFP, GFP alone, FLAG-CHMP4b or FLAG-epitope alone with anti-FLAG or anti-GFP antibody. Consequently, we observed that the Core but not the NS5A could bind to FLAG-CHMP4b (Fig. 4D). However, the Core was not immunoprecipitated with anti-FLAG antibody using the lysate mixtures of HCV-JFH1-infected RSc cell lysates and 293FT cells expressing FLAG-epitope alone or FLAG-Alix (Fig. 4D). Furthermore, the Core was coimmunoprecipitated with CHMP4b-GFP but not GFP when lysate mixtures of HCV-JFH1-infected RSc cells and 293FT cells expressing CHMP4b-GFP or GFP alone were used (Fig. 4D). In contrast, we failed to observe the marked colocalization of HCV-JFH1 Core with Myc-tagged TSG101 in HCV-JFH1-infected RSc cells expressing Myc-TSG101 or endogenous Alix in HCV-JFH1-infected RSc cells (Fig. 5A). Thus, we concluded that the HCV Core was associated with CHMP4b. Finally, we examined the subcellular localization of HCV Core and Brox, a novel farnesylated Bro1 domain-containing protein, since Brox was recently identified as a CHMP4-binding protein [27]. In this context, the Core partially colocalized with GFP-Brox in 293FT cells coexpressing of HCV Core and GFP-Brox (Fig. 5B). Importantly, we observed similar partial colocalization in HCV-JFH1-infected RSc cells expressing GFP-Brox (Fig. 5C). On the other hand, the CHMP4b-GFP alone was diffused in the cytoplasm of uninfected RSc cells (Fig. 5C). To examine the potential role of Brox in HCV life cycle, we established the Brox knockdown RSc cells by lentiviral vector expressing shRNA targeted to Brox (Fig. 5D). Consequently, we found that the release of HCV Core or the infectivity of HCV into the culture supernatants was significantly suppressed in the Brox knockdown cells 4 days after HCV-JFH1 infection (Fig. 5E and 5F), while the RNA replication of HCV-JFH1 was marginally affected in the knockdown cells (Fig. 5G) in spite of the very effective knockdown of Brox mRNA (Fig. 5D), suggesting that Brox is also required for the infectious HCV production.

## Discussion

In this study, we have demonstrated that the ESCRT system is required for infectious HCV production, and that HCV Core but not NS5A binds to CHMP4b, a component of ESCRT-III. Although RNA replication of HCV-JFH1 was not affected in the TSG101 knockdown or the Alix knockdown cells, the infectivity of HCV in the culture supernatants was significantly suppressed in these knockdown cells after HCV-JFH1 infection (Fig. 1G and 1H). Furthermore, siRNA targeted to TSG101, Alix, Vps4B, or CHMP4b significantly suppressed HCV Core level or the infectivity of HCV in the culture supernatants (Fig. 1L, 1M, and 2D), while the siRNA did not affect intracellular HCV Core level (Fig. 2E and 2F). Accordingly, we noticed some discrepancy that shRNA targeted to Vps4B or CHMP4b somewhat decreased intracellular HCV RNA replication (Fig. 1I), whereas siRNA targeted to TASG101, Alix, Vps4B or CHMP4b did not affect the RNA replication of subgenomic replicon of JFH1 (Fig. 2A and 2B). Furthermore, siRNAs targeted to TSG101, Alix, Vps4B, or CHMP4b did not affect HCV-O (genotype 1b) RNA replication using the OR6 assay system [17], [18] (Fig. 2C), indicating that the ESCRT system is unrelated to the HCV RNA replication of genotype 1b. Thus, we suggested that the ESCRT system is not significant for HCV RNA replication. Within the family *Flaviviridae*, NS3 of the Japanese encephalitis virus (JEV) is also known to interact with TSG101 and microtubules, suggesting their potential roles in JEV assembly [28]. Accordingly, HCV NS3 has been involved in HCV particle assembly and infectivity [29], [30]. However, whether HCV NS3 or another HCV protein binds to TSG101 remains to be investigated. At least, we did not observe the interaction between HCV NS5A and CHMP4b (Fig. 4D), Alix (Fig. 4D), TSG101, or Vps4B (data not shown).

Efficient enveloped virus release requires *cis*-acting viral late domains (L-domains), including P(T/S)AP, YPXnL (where X is any amino acid), and PPXY amino acid L-domain motif that are found in the structural proteins of other enveloped viruses [10], [11]. Unlike the case with these enveloped viruses, we failed to find the three types of conserved viral L-domain motif in the HCV-JFH1 Core (data not shown). Nevertheless, we observed that the Core bound to CHMP4b, whereas NS5A did not (Fig. 4D), suggesting that the Core has a novel motif required for the HCV production. In this regard, Blanchard *et al*. reported that the aspartic acid at position 111 in the Core is crucial for virus assembly [31]. Interestingly, the conversion of the aspartic acid into alanine at amino acid 111 (PTDP to PTAP), which creates the PTAP L-domain motif, enhanced the release of HCV Core in the cell culture medium (a 2.5-fold increase) compared with the wild-type Core [31]. In contrast, Klein *et al*. demonstrated that the D111A mutation in the Core had no effect on HCV capsid assembly [32]. Furthermore, Murray *et al* found that serine 99, a putative phosphorylation site, in the Core was essential for infectious virion production [7]. In any event, the Core motif that is needed for interaction with the ESCRT components remains to be identified.

Ubiquitin modification of viral protein has been implicated in virion egress as well as in protein turnover. Indeed, the ubiquitin/proteasome system is required for the retrovirus budding machinery, since proteasome inhibition interferes with retroviral Gag polyprotein processing, release, and maturation. The ubiquitin modification of the HIV-1 p6 domain of Gag enhances TSG101 binding [12]. In the case of HCV Core, proteasomal degradation of the Core is mediated by two distinct mechanisms [33]–[36]. E6AP E3 ubiquitin ligase mediates ubiquitylation and degradation of the Core [34]. In contrast, proteasome activator PA28γ (11S regulator γ), an HCV Core-binding protein, is involved in the ubiquitin-independent degradation of the Core and HCV propagation [33], [35], [36]. However, little is known whether or not the ubiquitin modification of the Core might be involved in HCV egress like other enveloped viruses.

Finally, the identification of the site of viral particle assembly and budding is an intriguing issue. In case of HCV, at present, it is very difficult to visualize the HCV budding site in the infected cells by an electron microscopy. However, recent studies suggested that lipid droplets are an important cytoplasmic organelle for HCV production [4]. In this regard, Shavinskaya *et al*. demonstrated that the lipid droplet-binding domain of the Core is a major determinant for efficient virus assembly [6]. The HCV Core induces lipid droplet redistribution in a microtubule- and dynein-dependent manner, since disrupting the microtubule network reduced the HCV titer, implicating transport networks in virus assembly and release [2]. Furthermore, Sandrin *et al*. reported that HCV envelope proteins localized to ESCRT-associated multivesicular body (MVB) [37]. Quite recently, Corless *et al*. have demonstrated that Vps4B and the ESCRT-III complex are required for HCV production [38]. Consistent with our findings, their dominant-negative forms of Vps4B and CHMP4B clearly suppressed HCV production. However, their dominant-negative forms of TSG101 and Alix failed to suppress the HCV production and they suggested that both TSG101 and Alix are unrelated to the HCV production. In contrast, we have demonstrated both TSG101 and Alix are also required for the infectious HCV production by using shRNAs and siRNAs (Fig. 1). We may partly explain such discrepancy due to the difference of the methodology in our study utilized shRNAs and siRNAs instead of dominant-negative forms of TSG101 and Alix. Importantly, they demonstrated that the dominant-negative forms of Vps4B and CHMP4b did not affect the HCV RNA replication and the accumulation of intracellular infectious particles, suggesting that Vps4B and CHMP4b are unrelated to HCV RNA replication. Indeed, we have demonstrated that the ESCRT system is not required for the assembly of intracellular infectious HCV particles (Fig. 2E). Notably, we have demonstrated for the first time that HCV Core associated with CHMP4b (Fig. 4). Accordingly, we have also demonstrated that Brox, a CHMP4b binding protein, is required for HCV production (Fig. 5D–G). Taking together the past and present findings, we propose that the ESCRT system is involved in infectious HCV production after the HCV assembly that occurs on lipid droplets.
